# Will You Accept the Government's Friend Request? Social Networks and Privacy Concerns

**DOI:** 10.1371/journal.pone.0080682

**Published:** 2013-11-27

**Authors:** David A. Siegel

**Affiliations:** Department of Political Science, Duke University, Durham, North Carolina, United States of America; Cinvestav-Merida, Mexico

## Abstract

Participating in social network websites entails voluntarily sharing private information, and the explosive growth of social network websites over the last decade suggests shifting views on privacy. Concurrently, new anti-terrorism laws, such as the USA Patriot Act, ask citizens to surrender substantial claim to privacy in the name of greater security. I address two important questions regarding individuals' views on privacy raised by these trends. First, how does prompting individuals to consider security concerns affect their views on government actions that jeopardize privacy? Second, does the use of social network websites alter the effect of prompted security concerns? I posit that prompting individuals to consider security concerns does lead to an increased willingness to accept government actions that jeopardize privacy, but that frequent users of websites like Facebook are *less* likely to be swayed by prompted security concerns. An embedded survey experiment provides support for both parts of my claim.

## Introduction

Technological progress brings new challenges to privacy, potentially spurring evolution of individuals' views toward privacy. At the individual level, an ever-expanding array of goods and services is offered at the sole cost of personal information. At the aggregate level, governments seeking intelligence against asymmetric threats such as terrorism increasingly ask–or order–citizens to give up privacy in the name of security. How do these two anti-privacy trends interact in shaping individuals' views toward privacy and governmental monitoring? I focus one particular aspect of individuals' privacy views that has come into sharp relief as governments seek to obtain intelligence to combat asymmetric threats: the way in which governments prompt individuals to consider security concerns so as to garner support for privacy-reducing governmental monitoring. Specifically, I show via an embedded survey experiment that while prompting individuals to consider security concerns can sway individuals toward greater support of governmental monitoring, frequent social network website use provides a bulwark against the anti-privacy effects of this prompt. This suggests changes in both government strategy and privacy protections over time as social network website use increases. My focus on individuals' views toward privacy and governmental monitoring provides a complement to an existing literature that is largely focused on legal privacy protections [1]–[4] and technical mechanisms to reduce threats to privacy [5].

I offer and test two hypotheses in this paper. First, I argue that framing policies that compromise individual privacy as “anti-terrorism” will garner higher levels of support than when these same policies are presented without such a frame. This threat frame is designed to offer a strong security-based rationale for governmental action by reminding individuals of extant threats in order to prompt them to consider security concerns. In this case the implicit threat is higher levels of terrorism. Salient security threats, e.g., in the aftermath of 9/11, have been previously correlated with greater acceptance of governmental monitoring [6], and threats in general have been shown to lead to a willingness to accept diminished civil liberties [7]. The threat frame might cause some individuals to feel that terrorism is an important reason to sacrifice privacy and permit governmental monitoring [8] or to remember information about the effect of data-gathering policies on the likelihood of terrorism [9], [10]. Either way, I expect that framing the policy as terrorism prevention should make individuals less opposed to governmental monitoring. I focus on internet surveillance by the government in this paper, as it is an area directly related to the concerns of users of social network websites. This leads to my first hypothesis:

### H1: Prompting security concerns increases individual support for governmental monitoring of the internet

However, prompting security concerns might be differentially effective based on exposure to the internet. I use the rise of social network websites over the past decade to help us understand this possibility. Over 900 million active users of Facebook alone voluntarily share private information not only with select groups of friends, but also with the company itself, despite the possible costs they might incur in doing so. Once shared, there is little preventing the company from passing information along to governments upon request, making monitoring simple and more encompassing. Given this, I would expect frequent use of social network websites to affect privacy views, and specifically responses to prompted security concerns, but how?

There are two broad ways of thinking about how the frequent use of social network websites affects an individual's attitudes toward privacy. The first is characterized by the following quote by Mark Zuckerberg (the founder of Facebook) in reference to the importance of online privacy: “[p]eople have really gotten comfortable…sharing more information…[t]hat social norm is just something that has evolved over time” [11]. In line with this quote, many observers now argue that the individuals who join social network websites express less concern for their privacy overall (e.g., refs. [12]–[15]). This viewpoint has some face validity, in that social network use requires divulging personal information which raises the specter of potential costs. As just a few examples of such costs in everyday life, speaking out against an employer can result in lost jobs (e.g., refs. [16], [17]), and sensitive postings can negatively affect divorce proceedings [18] or the chance of winning elected office [19]. Further, government's ability to request and be granted access to information posted on these websites offers additional potential costs: statements made or images posted might be taken to indicate harmful intent or potential unlawful activity. Accordingly, observers focused on these sorts of costs may implicitly view social network sites as a screening mechanism: individuals who do not want to divulge private information do not join or sharply limit their use when they do join, while those who join and are more frequent users are less concerned about their privacy. Were this screening mechanism dominant, I would expect that frequent users of social network websites would be less thoughtful about privacy concerns, and be more likely to be swayed by prompted security concerns.

In contrast, it might instead be the case that frequent users of social network websites are quite thoughtful about privacy and concerned about the potential costs of a loss of privacy, yet choose to share information anyway in order to garner the benefits of doing so. Among other things, these benefits include an increase in valuable social capital [20], [21]. Such users will be cautious in their approach to social network websites, carefully choosing which information to make public [22], [23] in order to achieve these social benefits at the lowest cost. The assumptions underlying this behavior match those typically associated with rational choice. Under this logic, frequent users of social network websites, aware of the potential costs of a reduction in privacy and suffering additional costs themselves due to more available personal information, will pay extra attention to the distribution of personal information and seek to minimize its uncontrolled release. They will find it cost effective to engage with complex privacy settings, and this experience may further increase the salience of privacy concerns. Frequent social network website users should be less likely to be swayed by prompted security concerns in this way of thinking.

I view this rational choice argument as more compelling than the screening argument. Frequent social network website users' engagement with privacy concerns, due to their having made the conscious choice to reveal private information, should imply that they are less likely to reconsider their views in response to prompted security concerns and less likely to view these security concerns as sufficient to overcome any existing worries about potential costs of anti-privacy policies. This leads to my second hypothesis:

### H2: The effect of exposure to prompted security concerns on individual support for governmental monitoring of the internet decreases as social network website use increases

Hypothesis 2 represents a crucial contribution of this paper. Whereas other studies have simply polled users of social network websites regarding privacy attitudes (e.g., ref. [24]), my survey experiment includes both users and non-users. This allows us to gauge the effect of social network website membership on one's response to prompted security concerns. Note that hypothesis 2 concerns only the conditional effect of social networking website use on one's response to prompted security concerns. I make no claim at all about any possible direct effect of social networking website use on views toward governmental monitoring.

## Results

I conducted a survey experiment to test my two hypotheses. My experimental design featured a treatment and a control group. The control group was asked the question “Do you agree or disagree with the following government actions…Monitoring internet usage?” while the treatment group was asked “Do you agree or disagree with the following government actions in an effort to prevent terrorism…Monitoring internet usage?” The control group's question provided little obvious upside for a respondent's acceptance of governmental intrusion into what is viewed as a largely private activity, and so yielded a baseline for views on privacy and governmental monitoring. I expected widespread disagreement with government's actions in this case. Further, I believe this scenario is one that has the possibility to elicit a reasoned response from all individuals queried, not just those with well-developed views on the subject. In contrast, the treatment group's question uses the specter of terrorism to provide a frame in which there is a strong security-based rationale for government action. This frame must ameliorate expected disagreement with the policy to have an effect, and so a significant effect of the frame on opinion would suggest an important role of prompted security concerns in driving privacy opinion. In addition, the frame is clearly tied to the context of national security policy, giving it extra relevance. In sum, the survey experiment's treatment evaluates the effect of a threatening, pro-security frame on the willingness of respondents to allow privacy intrusions by the government, in an area directly related to the concerns of users of social network websites and in the absence of a proximate threat.

**Figure 1:**
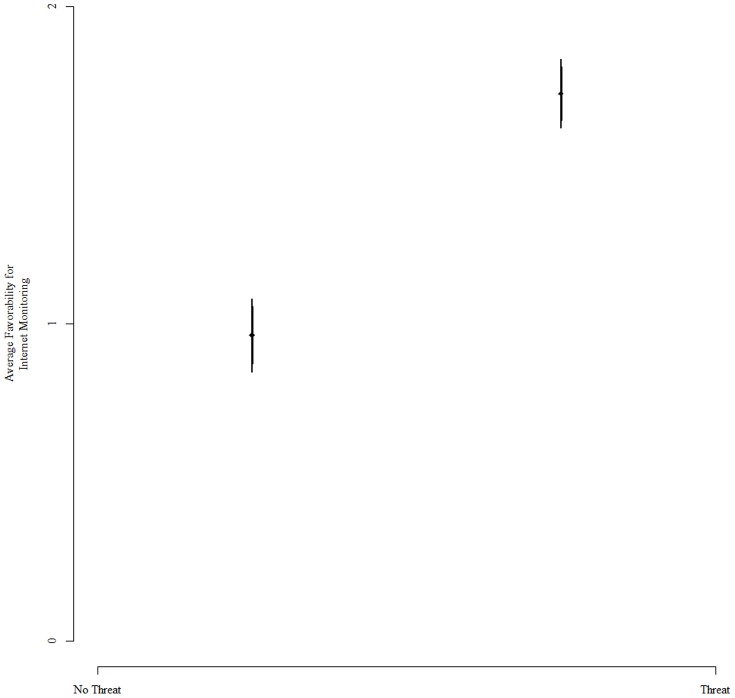
This figure displays the effect of the threat treatment on an individual's support for governmental monitoring of the internet (larger values imply greater support). The figure indicates that the treatment significantly increases support for these policies (p<.001). This result coincides with my theoretical expectations, indicating that mentioning threats makes policies purported to reduce threats more attractive.

Figure 1 displays the mean support for governmental monitoring of the internet for the treatment (i.e., with the threatening frame) and control groups; higher values indicate more support for the government program. It illustrates strong support for my first hypothesis. While resistance to governmental monitoring of internet usage was strong among all individuals in the control group, individuals in the treatment group expressed significantly more favorability. The difference between treatment and control was not only statistically significant (p<.001), it was substantively significant, increasing support for governmental monitoring by more than three-quarters of a point on a five point scale.

I next analyzed the heterogeneity in the degree of effectiveness of the threat frame in order to test my second hypothesis. To do so I used a regression framework to estimate the change with social network website use in the marginal effect of the treatment on opinions regarding governmental monitoring. Table 1 displays the results of this analysis. I note first that the treatment is effective in promoting support of governmental monitoring of internet behavior on average, and particularly so for individuals who are infrequent users of social network websites. The coefficient on the treatment indicates that it increases favorability toward governmental monitoring of individuals who never use social network websites by 0.816 points on a five point scale, a very large change. Though it was not one of my hypotheses, I also note that the use of social network websites in itself does not significantly alter individuals' general disapproval of internet monitoring by the government. In other words, absent the treatment, social network website use does not have a significant effect on one's opinion about governmental monitoring.

**Table 1:** This table displays the results of a regression including random effects for the respondent's state.

Variable	Coefficient	Standard Error	p-value (one-tailed)	t-statistic
Intercept	0.980	0.087	0.000	11.249
Social Network User	0.001	0.062	0.506	0.016
Treatment	0.816	0.106	0.000	7.699
Interaction	−.150	0.087	0.043	−1.713
Sigma(state)	0.088			
N	907			
LogLikelihood	−1607.016			
BIC	3254.892			

The dependent variable is support for governmental monitoring of the internet. The p-values represent one-tailed tests of the hypotheses of interest. The coefficient for the independent variable *Treatment* addresses Hypothesis 1, while the coefficient for the independent variable *Interaction* addresses Hypothesis 2.

The key variable in testing my second hypothesis, however, is the multiplicative interaction term Social Network User*Treatment. The coefficient of this variable indicates the degree to which being a social network website user changes the effect of the threat treatment on support for governmental monitoring of the internet. Table 1 indicates that the coefficient is negative and statistically significant at conventional alpha levels (one-tailed (due to the directional hypothesis) p<.05 or two-tailed p<.1), implying that frequent social network website users in the survey responded less strongly than non-users to prompted security concerns (i.e., the threat frame). This result is consistent with the idea underlying the second hypothesis: frequent users have already rationally considered potential privacy losses in their use of Facebook and other websites and are less open to altering beliefs about governmental monitoring based on a stated threat to security. In contrast, a positive coefficient on this term would not have been consistent with this idea. Instead, it would have implied that frequent social network website users responded more strongly to the threat frame, consistent with the idea that social network websites like Facebook screen for individuals who are less concerned about privacy and more open to reconsidering their positions on governmental monitoring in light of security concerns.

**Figure 2:**
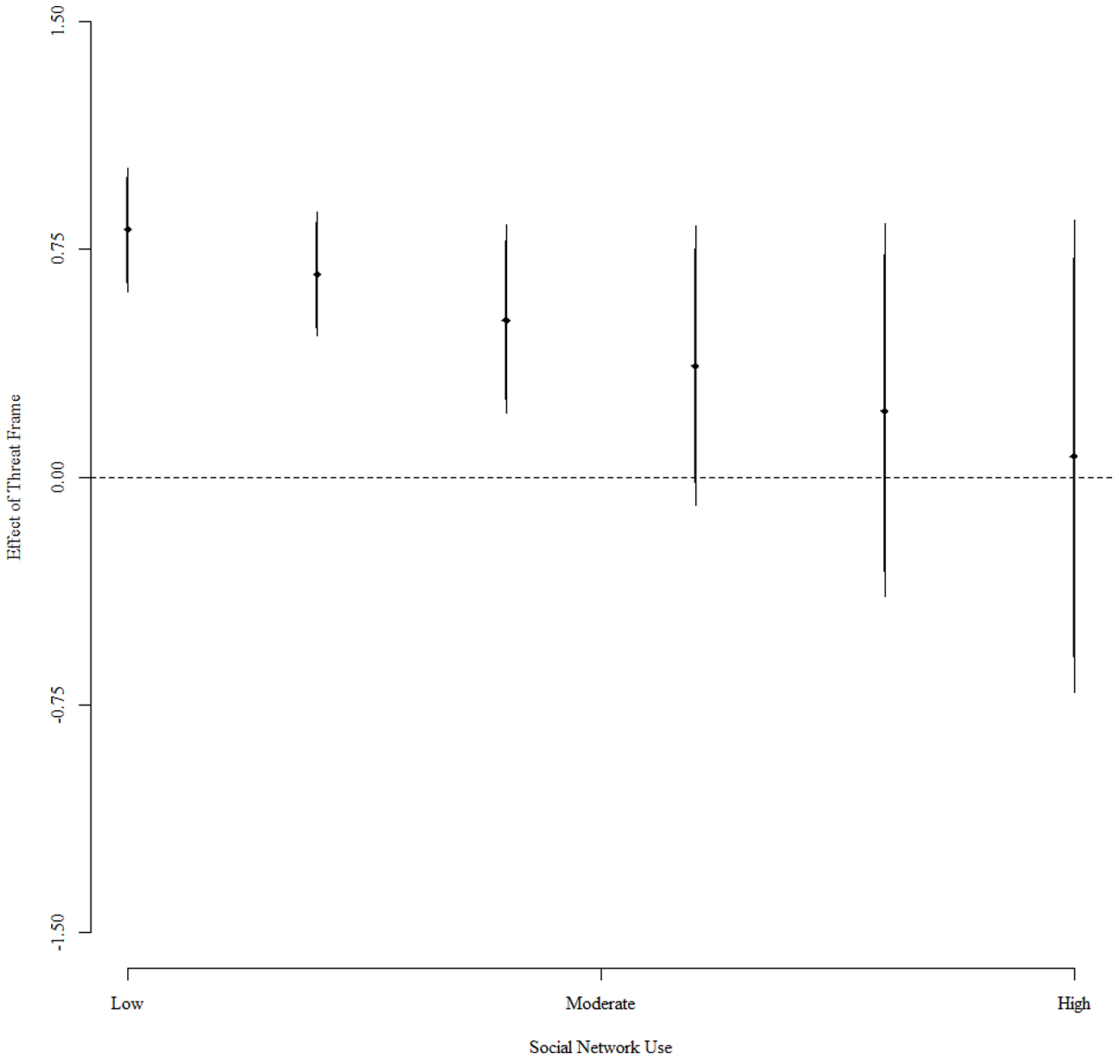
This figure displays the effect of the threat treatment for different levels of social network use (positive values indicate greater support for security policies). Each point estimate (represented by a diamond) shows the predicted change in an individual's level of support for government monitoring of the internet. Around each point estimate there are two lines. The heavier shaded line represents a 90% confidence interval, while the lighter shaded line represents a 95% confidence interval. The figure illustrates that the effect of the security treatment diminishes as social network website use increases. This supports the hypothesis that increased social network website use leads to more resistance to security-based rationales for increasing governmental monitoring.

Figure 2 illustrates the substantive significance of this result by displaying the effect of the treatment for different levels of social network website usage. The figure makes clear that frequently using social network websites does not just diminish the effectiveness of the treatment, it virtually eliminates it. The effect of the threat treatment declines as individuals increasingly utilize social network websites, and among the most frequent users the effect is close to and statistically indistinguishable from zero. In contrast, the effect of the security treatment is statistically significant and substantively meaningful for individuals who do not use social network websites. For these individuals, as I have noted, the security treatment substantially increases the probability that they agree with governmental monitoring of the internet. This means that frequent social network website use provides a bulwark against the government's otherwise useful tactic of prompting security concerns to sway individuals toward greater agreement with privacy-reducing governmental monitoring of the internet.

Though these results provide support in favor of the second hypothesis, I must note that they cannot prove with certainty that additional thoughtfulness regarding privacy concerns on the part of frequent social network users is the causal mechanism underlying the results. To more concretely address this question in future work one would need measures getting at how much each survey participant actually has thought about privacy, and preferably measures that are not self-reported statements about one's thoughtfulness. One potential survey instrument would be a battery of questions linking perceived levels of privacy risk to the true privacy risk individuals already experience. The further the perceived risk differs from the true risk, the less likely it is the individual has thought substantially about privacy concerns. An example of a question in such a battery is “Which of the following sets of information may Facebook share with third-parties as a default?” This battery should occur after the survey quasi-experiment in any new survey experiment in order to avoid priming individuals.

## Discussion

The use of social network websites exposes individuals to potential risks, many of which are observable in everyday life. In April 2011, for instance, a football coach was fired for posting negative comments about school officials on his Facebook account. This sort of story underlies the common wisdom that frequent use of social network websites such as Facebook is associated with decreased privacy concerns. After all, why would anyone concerned with privacy willingly suffer these potential costs? From this conclusion, it is easy to decide that frequent social network website users would be more willing to cede privacy to the government when prompted to do so by a security threat. My results suggest a possible gap in this logic: there are also benefits to the use of social network websites, and individuals' behavior might be driven by awareness of both costs and benefits of use. Such individuals may have their views less easily swayed by security threats due to this greater awareness of the issues involved in anti-privacy policies.

My results thus have clear policy implications. The explosive growth in social network website usage suggests that more individuals will be forced to work out their views on privacy, including governmental monitoring. The results from my study suggest that this trend signals greater resistance to government policies designed to trade decreased privacy for increased security, implying a significant future roadblock to the passage of anti-privacy policies.

## Methods

### Data

The October 2008 wave of the Cooperative Campaign Analysis Project (CCAP) contained this survey experiment. Replication data and other materials may be found at an author's public website: http://people.duke.edu/~das76. These data include numerous instruments taken from the CCAP, including party identification, gender, income, age, education, and geographic location, and can be used to help scholars answer questions regarding social network website use as related to demographic characteristics, or views on governmental monitoring as related to either social network use or demographic characteristics. The CCAP is an internet survey administered by YouGov Polimetrix, which draws from a large opt-in panel and constructs a sample via a sample matching methodology [25]. This experiment, which was approved by the Internal Review Board at Florida State University, includes 883 registered voters in the United States. Each survey was administered online, lasted between fifteen and twenty minutes, and included approximately forty questions. Two questions were specific to this analysis. The first asked for the amount of time spent on social network websites: “*On a typical day, about how much time do you spend on social networking websites (such as Facebook, MySpace, or LinkedIn)? <1>0 minutes <2>1–14 minutes <3>15–29 minutes <4>30–59 minutes <5>1 hour to under 2 hours <6>2 hours or more*”. A second question queried respondents' views on the government's “Monitoring Internet Usage.” Half of the sample, chosen randomly, was asked: “*Do you agree or disagree with the following government actions:…Monitoring Internet Usage?*” The second half was asked: “*Do you agree or disagree with the following government actions taken in an effort to prevent terrorism:…Monitoring Internet Usage?*” In each case the possible responses were “*<1> Strongly disagree <2> Disagree somewhat <3> Neither agree nor disagree <4> Agree somewhat <5> Strongly agree.*” In analyzing data from these variables I converted them to ordinal scales ranging from 0–5 and 0–4, respectively. For descriptive statistics on all variables included in the regression reported in table 1, see table 2. This table shows that my variables follow expectation. For instance, in 2008 Pew found that 29% of adults used online social networking [26]. In my sample, 27.6% of those surveyed reported using social networking websites. Specifically, 640 individuals reported not using social networks, 120 reported 1–14 minutes, 44 reported 15–29 minutes, 30 reported 30–59 minutes, 28 reported 1–2 hours, and 13 reported 2 hours or more. Please note that these descriptive statistics do not include imputed values, so the total number of individuals listed here will be less than used for my statistical modeling.

**Table 2:** This table displays descriptive statistics for the variables included in the regression model reported in Table 1.

Variable	Mean	Minimum	Maximum
Treatment	.539	0	1
Social Network Use	.543	0	5
Support for Monitoring of Internet Activity	1.375	0	4

This table displays the mean, minimum, and maximum values for each variable. These statistics were generated before imputation. Imputation does not substantively alter the distribution of these variables.

### Analysis

Due to random assignment to the treatment, to test the first hypothesis I used a simple t-test comparing means in the control and treatment groups. For the second hypothesis I estimated a multilevel regression with random effects for the respondent's state. This analysis utilized three explanatory variables in explaining support for the government's “Monitoring Internet Usage.” *Treatment* is coded one if the respondent received the security treatment, zero otherwise. *Social Network User* is an ordinal variable ranging from zero to five. A score of zero indicates that the individual does not use social network websites, while a score of five indicates that the individual uses these websites for more than two hours a day. This variable is not part of my hypotheses, and was included in the regression only because it is a necessary constituent term. Finally, I included the product term (Treatment*Social Network User) which estimated the effect of the treatment across social network website usage. I included no other variables because I had no prior belief–and found no suggestion in the literature–that any other variable should affect my results. Note that this would not have been true had I been interested in the direct effect of social network use on governmental monitoring, as the frequency of use of social network websites and views on privacy may systematically differ according to various demographic factors. But the same is not obviously true for the effect of social network website use on the marginal impact of the treatment on favorability toward governmental monitoring.

Despite this, I utilized several robustness checks. First, I included random effects in the regression to account for the possibility of regional similarities in the effect of social network website use on the treatment effect. I also ran models with a spate of demographic controls to address more fine-grained similarities; the results did not change substantively. To account for missing data in the reported use of social network websites I implemented multiple imputation, creating 5 multiply imputed datasets and averaging across these datasets. The results do not qualitatively change if I do not impute the data. I used various demographic variables (e.g. age, income, education) in the imputation model, which treated variables as continuous.

## References

[1] Slobogin C (2007) Privacy at risk: The new government surveillance and the Fourth Amendment. University of Chicago Press.

[2] BeVier LR (1995) Information about individuals in the hands of government: Some reflections on mechanisms for privacy protection. Wm. & Mary Bill Rts. J. 4: 455.

[3] Westin AF (2003) Social and Political Dimensions of Privacy. Journal of Social Issues 59: 431–453.

[4] Sundby SE (1994) “Everyman's” Fourth Amendment: Privacy or Mutual Trust between Government and Citizen? Columbia Law Review 94: 1751–1812.

[5] Goldberg I, Wagner D, Brewer E (1997) Privacy-enhancing technologies for the Internet. Compcon' 97. Proceedings, IEEE 103–109.

[6] Harris Interactive, Westin A (2001a). The Harris Poll: #49, New York City (survey report).

[7] Doty RM, Peterson BE, Winter DG (1991) Threat and authoritarianism in the United States, 1978–1987. Journal of Personality and Social Psychology 6: 629–640. 10.1037//0022-3514.61.4.629 1960654

[8] Nelson TE, Oxley ZM (1999) Issue Framing Effects on Belief Importance and Opinion. The Journal of Politics 61: 1040–1067.

[9] Kinder DR, Sanders LM (1996) Divided by Color: Racial Politics and Democratic Ideals. Chicago: University of Chicago Press.

[10] Chong D, Druckman JN (2007) Framing Theory. Annual Review Political Science 10: 103–126.

[11] Johnson B (2010) Privacy No Longer a Social Norm, Says Facebook Founder. The Guardian. Available: http://www.guardian.co.uk/technology/2010/jan/11/facebook-privacy. Accessed 2013 Oct 20.

[12] Livingstone S (2008) Taking Risky Opportunities in Youthful Content Creation: Teenagers' Use of Social Networking Sites for Intimacy, Privacy, and Self-Expression. New Media & Society 10: 393–411.

[13] Resinger D (2009) Study: Facebook Users Willingly Give Out Data. CNET. Available. : http://news.cnet.com/8301-17939_109-10410257-2.html?tag=newsEditorsPicksArea.0. Accessed 2013 Oct 20.

[14] Nussbaum E (2007) Say Everything. New York Magazine 12 February. Available: http://nymag.com/news/features/27341/. Accessed 2013 Oct 20.

[15] Ducklin D (2009) Sophos Australia Facebook ID Probe 2009. Sophos 6 December. Available: http://nakedsecurity.sophos.com/2009/12/06/facebook-id-probe-2009/. Accessed 2013 Oct 20.

[16] Clarke J (2011) Fitton Fired for Facebook Rants. The Sydney Morning Herald 18 April.

[17] Scott W (2011) Brooke Coach Fired After Facebook Rant. 28 April. Available: http://www.theintelligencer.net/page/content.detail/id/554525.html. Accessed 2013 Oct 20.

[18] Mandel N (2011) Marriage Over? Divorce Lawyer Says Facebook is Used in 90 Percent of Cases. New York Daily News 2 May.

[19] Peters JW, Stelter B (2010) The Facebook Skeletons Come Out. The New York Times 5 November. Available: http://www.nytimes.com/2010/11/07/fashion/07indiscretions.html. Accessed 2013 Oct 20.

[20] Ellison NB, Steinfield C, Lampe C (2007) The Benefits of Facebook ‘Friends:’ Social Capital and College Students' Use of Online Social Network Sites. Journal of Computer-Mediated Communication 12: online.

[21] Boyd DM, Ellison NB (2008) Social Network Sites: Definition, History, and Scholarship. Journal of Computer-Mediated Communication 13: 210–230.

[22] Acquisti A, Gross R (2006) Imagined Communities: Awareness, Information Sharing, and Privacy on the Facebook. Lecture Notes in Computer Science 4258: 36–58.

[23] Holson LM (2010) Tell-All Generation Learns to Keep Things Offline. New York Times 8 May. Available: http://www.nytimes.com/2010/05/09/fashion/09privacy.html?hp. Accessed 2013 Oct 20.

[24] Boyd D, Hargittal E (2010) Facebook Privacy Settings: Who Cares? First Monday 15: 13–20.

[25] Rivers D, Bailey D (2009) Inference from matched samples in the 2008 US national elections. Proceedings of the Joint Statistical Meetings 627–639.

[26] Merino F (2011) Pew: Social networking sites hit new milestones. Vatornews 26 August. Available: http://vator.tv/news/2011-08-26-pew-social-networking-sites-hit-new-milestones. Accessed 2013 Oct 20.

